# The Story of the Insane from Year to Year

**Published:** 1890-10-11

**Authors:** 


### The Story of the Insane from Year to Year.

[Annual Reports, Pamphlets, &c., should be addressed to The Editor, The Lodge, Porchester Square, London, W.]

### NORTHUMBERLAND COUNTY ASYLUM.

At the close of the year 1860 there were only 161 patients in this asylum; now there are 543. One hundred and twenty-three cases were admitted during the year; 51 were discharged recovered, and 46 died. The percentage of recoveries on admissions was 41, while the deaths were 8·5 on the average number resident. Six of the deaths were owing to general paralysis, and not fewer than twelve to phthisis. The medical superintendent has given lectures on ambulance work—a praiseworthy example, which it is hoped will be extensively followed in other asylums. The Commissioners recommend the extension of the infirmary wards, and also the appointing of permanent night nurses in these wards. The weekly rate stands at 9s. 7½d. per head. The visitors state that the matron, after long and faithful service, retired from her post, and that they granted her a retiring allowance of £90 a-year. Dr. MacDowall says, when alluding to the matron's resignation, that it "would be ungrateful did he not officially acknowledge the conscientious, energetic, and successful manner in which she discharged her duties, and the creditable way in which the work of her department was always performed." While regretting that ill-health was the cause of Miss Turner's resignation, we congratulate her on receiving the pension, and join with Dr. MacDowall n the hope that she may long live to enjoy it.

